# Imaging features of right atrial lipoma on multimodality imaging: A case report

**DOI:** 10.1097/MD.0000000000050606

**Published:** 2026-09-11

**Authors:** Qilong Xie, Aijuan Fang, Xinmin Chen, Qimeng Wei, Qi Cai

**Affiliations:** a Bengbu Medical University Graduate School, Bengbu, China; b.Department of Ultrasound Medicine, Nanjing University Medical School Affiliated Nanjing Drum Tower Hospital, Nanjing, China; c.Xuancheng People’s Hospital, Affiliated Xuancheng Hospital of Wannan Medical College, Xuancheng, China.

**Keywords:** case report, lipoma, multimodality imaging, right atrium

## Abstract

**Rationale:**

Cardiac lipoma is the second most common benign primary cardiac tumor. It can arise in any cardiac chamber and often presents with no or only nonspecific symptoms. Its variable morphology poses a diagnostic challenge.

**Patient concerns:**

A 53-year-old asymptomatic female patient reported no history of palpitations, chest tightness, or other discomfort, and denied lower extremity edema or limitation of physical activity. During a routine health checkup, transthoracic echocardiography (TTE) revealed a mass at the junction of the superior vena cava and the right atrium. She then presented to our hospital for further evaluation.

**Diagnoses:**

Further evaluation with transesophageal echocardiography (TEE) revealed a right atrial mass with hypoechoic to isoechoic characteristics, measuring approximately 2.86 cm × 1.62 cm. Computed tomography demonstrated a well-circumscribed nodular lesion with fat attenuation within the right atrium. Positron emission tomography–computed tomography revealed minimal glucose uptake in the mass, with a maximum standardized uptake value of 1.3. Cardiac magnetic resonance imaging showed low signal intensity on T2-weighted fat-suppressed images. The diagnosis of a right atrial lipoma was ultimately established following surgical excision and histopathological confirmation.

**Interventions:**

The patient underwent cardiac tumor resection, and the mass was completely removed. Postoperative pathology confirmed it as a cardiac lipoma. The patient remained hemodynamically stable after surgery, and no recurrence has been observed during follow-up to date.

**Outcomes and lessons:**

This case highlights the complementary diagnostic value of TEE in conjunction with TTE and demonstrates that a comprehensive multimodality imaging approach improves diagnostic accuracy for cardiac lipomas and facilitates differentiation from benign myxomas and malignant liposarcomas.

**Take-home message:**

Right atrial lipoma is a benign tumor with variable morphological features. An integrated multimodality imaging approach is crucial for achieving a reliable diagnosis and guiding appropriate clinical management.

**Learning points**

Cardiac lipomas are soft, encapsulated masses composed of mature adipose tissue that may arise within the cardiac chambers, myocardium, or pericardium. Large lesions can impair cardiac hemodynamics or involve the conduction system, potentially resulting in malignant arrhythmias or even sudden cardiac death.

TEE provides incremental diagnostic value over TTE, particularly in the evaluation of small or anatomically complex intracardiac masses, which are often missed or misdiagnosed on TTE.

Multimodality imaging plays a pivotal role in the reliable diagnosis and differential diagnosis of cardiac lipomas.

## 1. Introduction

Cardiac lipoma is a relatively common benign primary cardiac tumor, ranking second in prevalence after cardiac myxoma. Previous studies have shown that cardiac lipomas may originate from the endocardium, epicardium, myocardium, or pericardium, with a predilection for the right atrium and left ventricle.^[1]^ These tumors can be diagnosed at any age and exhibit no significant sex predilection.^[2]^ Although cardiac lipomas are typically solitary lesions, rare cases involving multiple lipomas affecting both ventricles have been reported.^[3]^ Dyspnea is the most commonly reported symptom in patients with cardiac lipomas, followed by fatigue and palpitations.^[4]^ Larger lesions may cause arrhythmias through mechanical compression,^[5]^ and rare cases of left coronary ostial obstruction leading to sudden death have been reported.^[6]^ Therefore, surgical resection is generally recommended.

## 2. History of presentation

**Figure 1.**
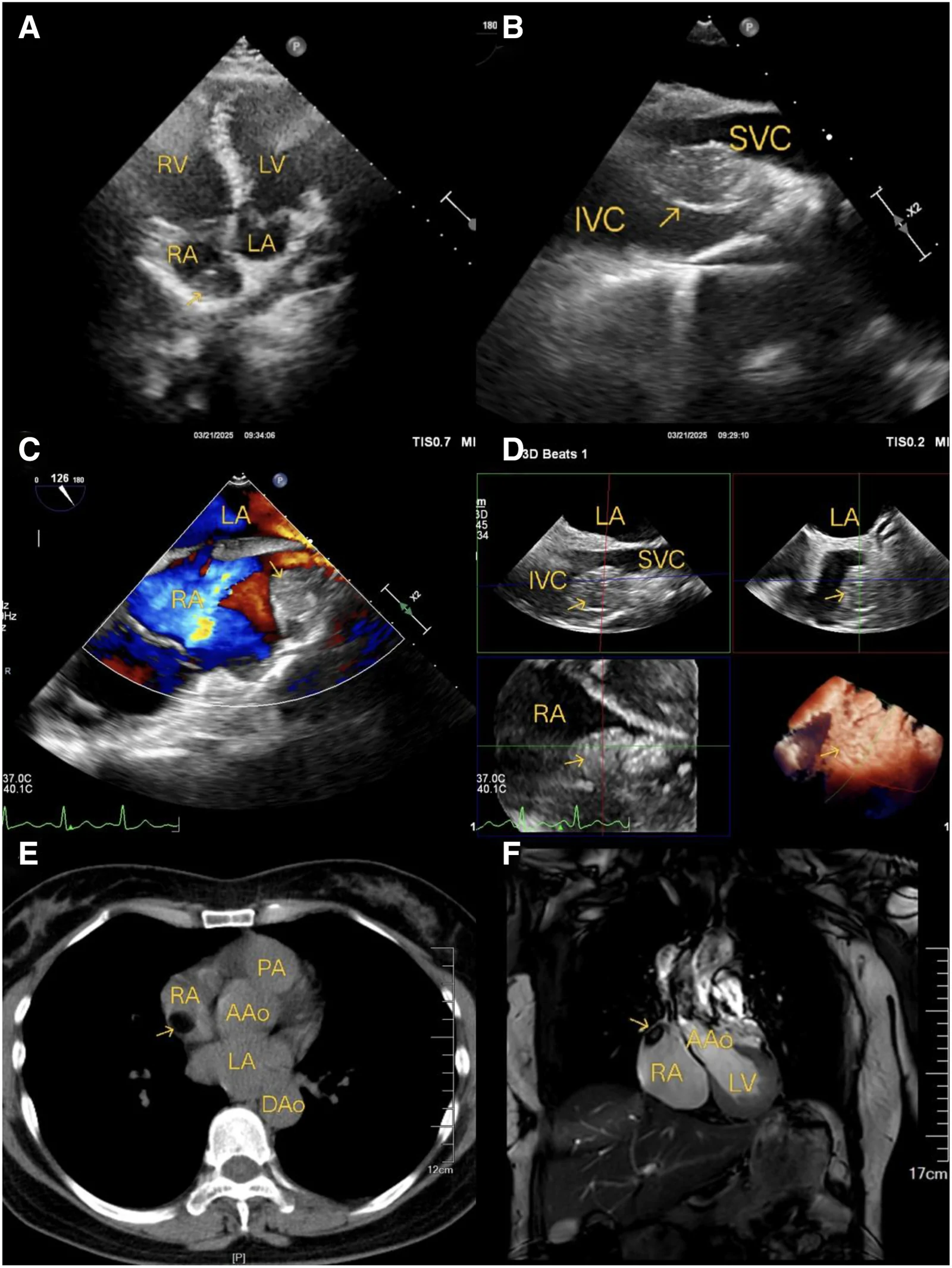
(A) Apical 4-chamber view on transthoracic echocardiography (TTE) showing a hypoechoic to isoechoic mass within the right atrium, adjacent to the orifice of the superior vena cava. (B-D) Two-dimensional and 3-dimensional transesophageal echocardiography (TEE) demonstrating a hypoechoic to isoechoic mass with well-defined borders at the superior vena cava-right atrial junction. The lesion appears pedunculated and shows no significant internal flow signal on Doppler imaging. (E) Mediastinal window on computed tomography (CT) revealing a relatively well-defined, fat-attenuation nodule located in the right atrium. (F) Cardiac magnetic resonance (CMR) imaging on a T2-weighted fat-suppressed sequence showing low signal intensity of the mass. The arrow indicates the location of the mass. AAo = ascending aorta; DAo = descending aorta; CMR = Cardiac magnetic resonance; CT = Computed tomography; IVC = inferior vena cava. LA = left atrium; LV = left ventricle; RA = right atrium; RV = right ventricle; SVC = superior vena cava; TTE = Transthoracic echocardiography.

The patient was asymptomatic, denying palpitations, chest discomfort, lower extremity edema, or reduced exercise tolerance. On admission, her vital signs were within normal limits, with a blood pressure of 135/85 mm Hg. Laboratory findings included a red blood cell count of 4.17 × 10^12^/L, B-type natriuretic peptide (BNP) of 16.7 pg/mL, procalcitonin (PCT) of 0.024 ng/mL, interleukin-6 (IL-6) of 2.41 pg/mL, high-sensitivity cardiac troponin T (hs-cTnT) of 0.005 μg/L, an erythrocyte sedimentation rate (ESR) of 9 mm/h (instrumental method), and a D-dimer level of 4.30 mg/L. Physical examination revealed no abnormalities. Transthoracic echocardiography (TTE) identified a hypoechoic to isoechoic lesion at the junction of the superior vena cava and the right atrium (Fig. 1A). Owing to the small size of the lesion and its visualization solely in the apical 4-chamber view, differentiation from an imaging artifact was challenging. Consequently, the patient was hospitalized for additional diagnostic assessment.

## 3. Past medical history

The patient had no past surgical history. She had no known history of hypertension or diabetes mellitus and reported no family history of extracardiac malignancy or venous thromboembolism.

## 4. Investigation by multimodality

**Figure 2.**
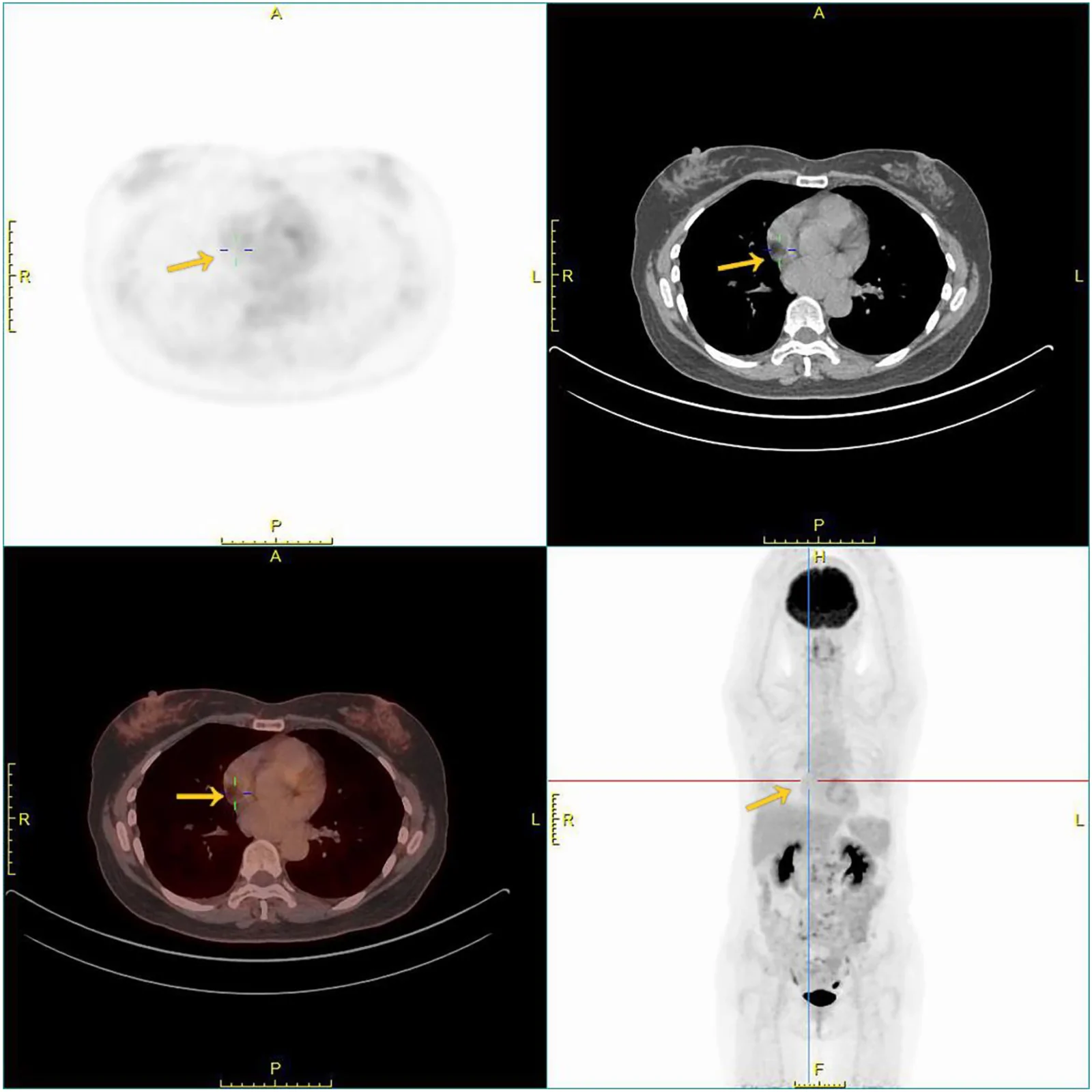
The PET/CT fusion image demonstrated reduced FDG uptake within the lesion (SUVmax = 1.3), in contrast to the physiological uptake observed in the surrounding myocardium. The arrow indicates the location of the mass. FDG = ^18^F-fluorodeoxyglucose, PET/CT = positron emission tomography/computed tomography, SUVmax = maximum standardized uptake value.

On admission, routine electrocardiography (ECG) demonstrated sinus rhythm with a normal tracing. Two-dimensional and 3-dimensional transesophageal echocardiography (TEE )identified an irregular mass at the orifice of the superior vena cava within the right atrium, measuring approximately 2.86 cm × 1.62 cm. The lesion exhibited a hypoechoic to isoechoic internal echotexture without detectable internal blood flow signals. It was encapsulated by a hyperechoic rim and appeared well circumscribed, pedunculated, and mobile. No significant obstruction of superior vena cava inflow was observed, with a peak flow velocity of approximately 1.0 m/s. Furthermore, there was no sonographic evidence of invasion into adjacent cardiac tissues or distant structures (Figures 1B, 1C, and 1D). These echocardiographic features were suggestive of a benign cardiac mass; however, definitive differentiation from a cardiac myxoma remained challenging based on echocardiography alone. Computed tomography (CT )demonstrated a well-defined fat-attenuation nodule within the right atrium, measuring approximately 16 mm in length (Fig. 1E).Cardiac magnetic resonance (CMR) imaging revealed a right atrial mass measuring approximately 21 × 10 mm, with low signal intensity on T2-weighted, fat-suppressed sequences, consistent with adipose tissue (Fig. 1F). Positron emission tomography-computed tomography (PET-CT) further identified a fat-attenuation lesion in the right atrium, measuring approximately 1.9 cm × 1.2 cm, with reduced radiotracer uptake and a maximum standardized uptake value of 1.3 (Fig. 2). Based on the collective findings from multimodality imaging, the diagnosis strongly favored a benign cardiac lipoma. Compared with the initial echocardiographic assessment, CT, CMR, and PET-CT provided significant incremental value in both the diagnosis and differential diagnosis of the intracardiac mass.

## 5. Management

**Figure 3.**
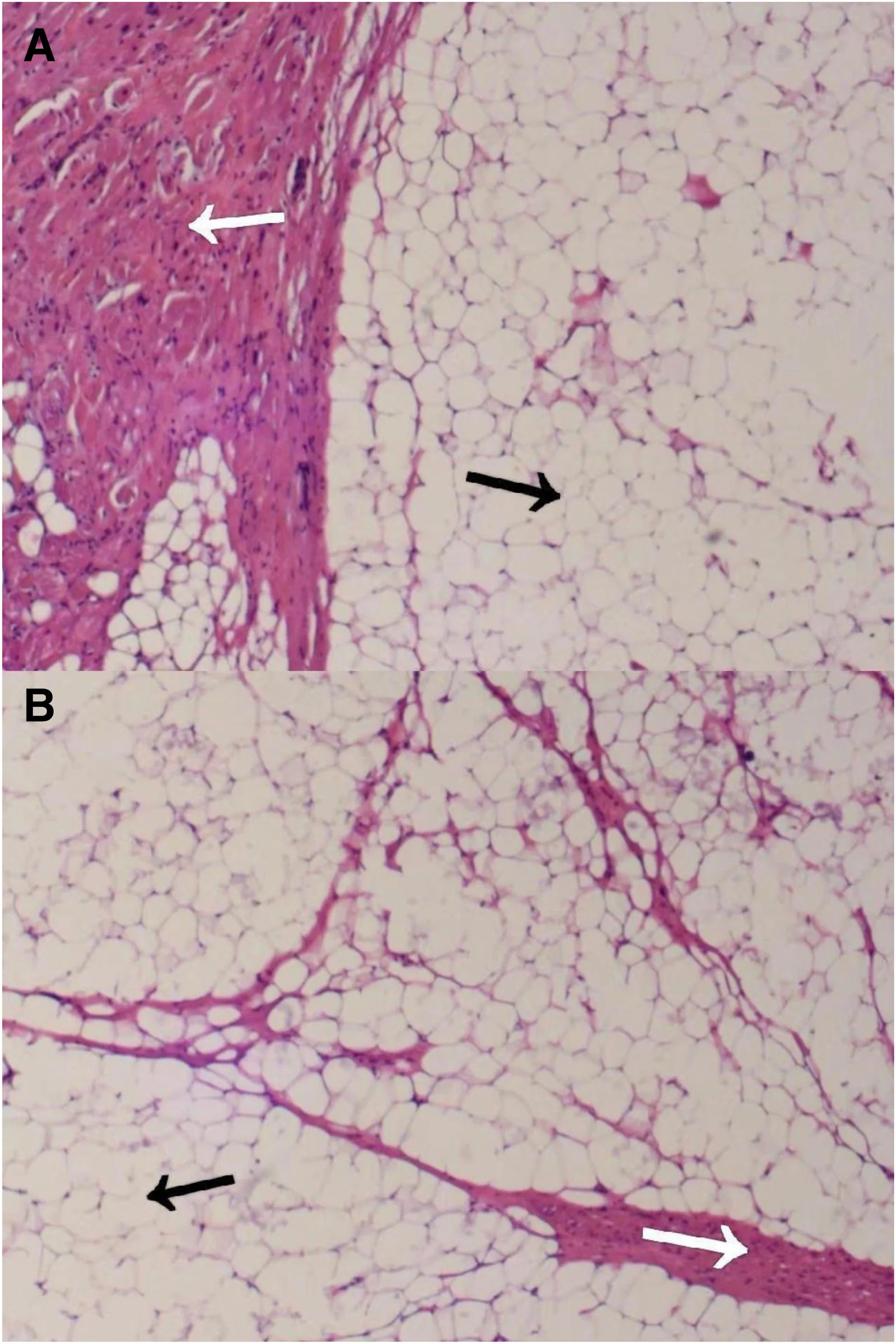
(A and B) Hematoxylin and eosin–stained histopathological sections of the resected specimen demonstrating normal cardiomyocytes interspersed with abundant mature adipocytes. White arrowheads indicate right atrial cardiomyocytes, and black arrowheads indicate adipocytes.

The patient underwent surgical resection of the cardiac tumor. Intraoperatively, the heart showed no significant enlargement. Direct intracardiac inspection revealed a pedunculated mass within the right atrium, with its stalk originating from the orifice of the superior vena cava. The tumor was completely excised. Histopathological examination confirmed the presence of abundant mature adipocytes adjacent to normal cardiomyocytes, consistent with a diagnosis of right atrial lipoma (Figs. 3A, 3B).

## 6. Outcome

Postoperatively, the patient remained hemodynamically stable. Follow-up TTE demonstrated no residual mass in the right atrium. Her postoperative recovery was uneventful, and she was discharged in good clinical condition. During 9 months of follow-up, there was no evidence of tumor recurrence.

## 7. Discussion and conclusion

TTE is the first-line, noninvasive imaging modality for evaluating cardiac masses, allowing assessment of size, morphology, echogenicity, mobility, and hemodynamic impact. In this case, TTE demonstrated a hypoechoic-to-isoechoic mass in the right atrium; however, visualization was limited to the apical 4-chamber view, providing insufficient information for definitive characterization. Subsequent TEE revealed a well-defined, pedunculated, and highly mobile hypoechoic mass near the superior vena cava orifice, without internal Doppler flow signals or significant obstruction of superior vena cava inflow. Thus, TEE complemented the limited field of view of TTE and provided incremental diagnostic value by enabling more detailed anatomical assessment. Nevertheless, TEE is limited in its ability to characterize tissue composition. In the present case, the echocardiographic features initially suggested a cardiac myxoma. Cardiac myxoma, the most common benign primary cardiac tumor, typically appears as a pedunculated, smooth-surfaced, and mobile mass on echocardiography,^[7]^ closely mimicking the TEE findings observed in this lipoma. Consequently, additional imaging modalities were required to establish a definitive diagnosis.

CT, with its high spatial resolution and rapid acquisition, enables accurate assessment of tumor internal density and invasive margins, facilitating lesion characterization. PET-CT provides both morphological and metabolic information, aiding in the differentiation between benign and malignant tumors and in the detection of distant metastases. Although PET-CT is well established for the evaluation of extracardiac lipomas, its role in cardiac lipomas remains limited due to their characteristically low metabolic activity.^[8]^ CMR, owing to its superior soft-tissue contrast, is a pivotal noninvasive modality for the assessment of cardiac masses and offers high diagnostic accuracy. Typical CMR features of cardiac lipomas include high signal intensity on T1- and T2-weighted images with marked signal suppression on fat-suppressed sequences, confirming the fatty composition of the lesion. In addition, cine CMR enables evaluation of hemodynamic effects, while myocardial infiltration can be sensitively detected. In the present case, PET-CT excluded metastatic involvement from an extracardiac malignancy and ruled out a primary malignant cardiac tumor such as liposarcoma, supporting the diagnosis of a benign primary neoplasm. Both CT and CMR confirmed the fatty nature of the mass. Notably, CMR demonstrates high specificity in differentiating liposarcoma based on characteristic features, including large tumor size, nonfatty components, high T2-weighted signal intensity, and thick internal septa.^[9]^ Collectively, the multimodality imaging findings led to a definitive diagnosis of benign cardiac lipoma.

This case highlights the value of multimodality imaging in diagnosing cardiac lipoma. A comprehensive imaging approach enables accurate assessment of tumor size, tissue characteristics, and depth of involvement, thereby ensuring diagnostic confidence. As no standardized treatment guidelines exist for cardiac lipomas, management should be individualized. In most cases, complete surgical excision is recommended because of the potential risk of progressive growth and myocardial infiltration.^[10]^ In the present case, the patient underwent successful tumor resection and has remained free of recurrence to date.

## Author contributions

**Conceptualization:** Qilong Xie, Xinmin Chen, Qimeng Wei.

**Investigation:** Qilong Xie, Xinmin Chen, Qimeng Wei.

**Methodology:** Aijuan Fang.

**Supervision:** Qi Cai.

**Writing – review & editing:** Qi Cai.

**Writing – original draft:** Qilong Xie, Aijuan Fang.
